# Organic Synthesis

**Published:** 1869-10

**Authors:** 


Organic Synthesis.—The last number of Liebig's *Annalen* announces the fact, that the direct transformation of the acids of the fatty series into corresponding alcohols has been effected by *Linnemann.* It was accomplished by the action of sodium-amalgam on anhydrous acid. This important discovery supplies the missing link required to pass step by step up the ladder from the simplest alcohol (wood-spirit), up to the highest, wax alcohol.—*British Med. Journal.*

